# Multi-scale dynamic graph neural network for PM2.5 concentration prediction in regional station cluster

**DOI:** 10.1371/journal.pone.0338392

**Published:** 2025-12-04

**Authors:** Xin Lu, Juyang Liao, Huihua Huang, Jiazhen Li, Hao Huang, Yingfang Zhu

**Affiliations:** 1 College of Computer Science and Mathematics, Central South University of Forestry and Technology, Changsha, China; 2 Hunan Changsha-Zhuzhou-Xiangtan City Cluster National Research Station of Ecosystem, Hunan Botanical Garden, Changsha, China; National University of Defense Technology, CHINA

## Abstract

Accurate prediction of PM2.5 concentrations is crucial for public health and environmental management. However, effectively capturing complex spatiotemporal dependencies across multiple time scales remains a persistent challenge for existing methods, particularly in regions with sparse monitoring stations. This study proposes a Multi-Scale Dynamic Graph Neural Network (MSDGNN) for PM2.5 forecasting in station clusters. The model incorporates multi-scale temporal modeling (hourly, daily, weekly) to capture both short- and long-term dependencies. A learnable mapping matrix dynamically groups stations to strengthen spatial correlation learning. Furthermore, MSDGNN employs multi-head attention and spatiotemporal graph attention mechanisms to construct dynamic graphs, utilizing adaptive adjacency matrices and Chebyshev graph convolutions for effective feature propagation. We evaluated MSDGNN on 22 air quality monitoring stations in the Chang-Zhu-Tan region. Results demonstrated that our model reduces MAE by 6.77% and RMSE by 8.67% compared to the best baseline, validating its capability to learn complex dependencies and deliver accurate predictions under diverse spatiotemporal conditions.

## Introduction

Amid rapid economic growth and extensive urban expansion, air pollution has emerged as a critical global environmental and public health challenge [1]. Particulate matter (PM), especially fine particulate matter PM2.5, is among the most harmful pollutants due to its ability to penetrate deeply into the human respiratory system [2]. Epidemiological studies indicate that for every 10 μg/m³ increase in PM2.5 concentration, cardiovascular mortality rises by approximately 11% [3]. Consequently, reliable prediction of PM2.5 levels is essential for safeguarding public health and mitigating adverse health impacts [4].

Current methodologies for PM2.5 concentration prediction primarily fall into two categories: numerical simulation-based approaches and data-driven models. Numerical simulation models [5–7], grounded in atmospheric dynamics, utilize emission inventories, meteorological data, and boundary conditions to simulate pollutant diffusion and chemical reactions [8]. However, these models often face limitations in capturing localized variations, particularly in regions with complex topography or heterogeneous pollution sources [9]. In contrast, Data-driven approaches learn directly from high-frequency, multi-dimensional sensor data, offering a powerful means to address complex regional characteristics [10].

Traditional machine learning techniques, such as Linear Regression (LR) [11], Random Forests (RF) [12], and Support Vector Regression (SVR) [13], have demonstrated improvements over classical statistical methods (e.g., Historical Average, ARMA [14], ARIMA [15]) in handling large datasets. Nevertheless, these methods often fail to adequately capture the temporal dependencies critical for accurate PM2.5 forecasting [16]. Deep learning models, including Convolutional Neural Networks (CNN) [17,18], Long Short-Term Memory networks (LSTM) [19,20], and Gated Recurrent Units (GRU) [21,22] effectively address temporal modeling challenges [23]. Despite these improvements, a common limitation of these approaches is their predominant focus on either spatial or temporal patterns, often at the expense of simultaneous multi-scale integration.

Unlike traditional CNNs and RNNs designed for Euclidean data, graph neural networks (GNNs) perform convolutions on irregular, non-Euclidean structures, offering flexible modeling of complex relationships between nodes [24]. This capability enables effective capture of spatial correlations in pollutant dispersion and multi-dimensional associative features [25]. For instance, Bai et al.[26] proposed an Adaptive Graph Convolutional Recurrent Network (AGCRN), which incorporates Node Adaptive Parameter Learning (NAPL) to generate node-specific parameters, thereby capturing spatial dependencies and site-specific characteristics. Li et al.[27] developed a Dynamic Graph Convolutional Recurrent Network (DGCRN) that employs hypernetworks to dynamically generate adjacency matrices at each time step, capturing dynamic spatiotemporal correlations. Choudhury et al.[28] proposed AGCTCN, combining spatial attention with graph and temporal convolutions to model spatiotemporal dependencies for short-term PM2.5 forecasting.

In parallel, Transformer-based architectures have gained prominence for sequence modeling, leveraging multi-head self-attention to process multiple feature subspaces in parallel and handle long-range dependencies. Liang et al.[29] proposed AirFormer, which incorporates Dartboard Spatial MSA and Causal Temporal MSA to model spatiotemporal dependencies for nationwide PM2.5 predictions. Zhang et al.[30] introduced Crossformer, which decomposes and aggregates multi-scale information through multi-head attention for comprehensive feature representations.

Despite these advancements, current GNN and Transformer-based models exhibit limitations in simultaneously capturing multi-scale spatiotemporal dependencies. Spatiotemporal data in PM2.5 prediction inherently exhibit multi-scale characteristics: spatially, from individual sites to regional clusters; temporally, from hourly fluctuations to weekly periodic patterns. However, existing GNN and Transformer-based models are inadequate for the effective capture of these cross-scale spatiotemporal dependencies. Furthermore, in practice, monitoring stations are often sparsely distributed due to economic and logistical constraints, resulting in weak data associations between distant stations. This sparsity leads to insufficient modeling of long-range spatiotemporal dependencies, hindering accurate capture of pollutant transport patterns from distant sources [31].

To address these challenges, we proposed a Multi-Scale Dynamic Graph Neural Network (MSDGNN) for PM2.5 forecasting. The model employs parallel multi-scale temporal modeling to capture both short-term fluctuations and long-term periodic patterns. Spatially, it utilizes a learnable grouping module that dynamically clusters stations based on functional similarities, enhancing long-range dependency modeling in sparse networks. The architecture integrates Multi-Head Attention for global spatial correlations and Spatiotemporal Graph Attention for dynamic relationship weighting. These features are processed through an Adaptive Graph Convolution module using Chebyshev approximation on a hybrid graph structure. Extensive evaluations on real-world data from 22 monitoring stations in the Chang-Zhu-Tan region demonstrate that our model significantly outperforms existing state-of-the-art methods.

## Data and problem statement

### Study area and data

The Chang-Zhu-Tan (CZT) urban agglomeration, situated in the middle reaches of the Yangtze River in China, encompasses the cities of Changsha, Zhuzhou, and Xiangtan. As a pivotal region within the Yangtze River Economic Belt, the CZT has experienced rapid economic growth and urbanization, leading to increasing challenges associated with PM2.5 pollution.

Hourly air quality data were collected from 22 monitoring stations across the three cities via the National Urban Air Quality Real-Time Release Platform (https://air.cnemc.cn:18007/). The dataset spans from January 2020 to December 2023 and includes measurements of six major pollutants (PM2.5, PM10, NO₂, CO, O₃, SO₂) as well as the Air Quality Index (AQI).

### Problem statement

PM2.5 prediction faces three interconnected challenges: capturing multi-scale temporal patterns, modeling spatial correlations between stations, and handling sparse monitoring networks. We formulate this as a spatiotemporal graph learning problem with dynamic station grouping.

#### Spatiotemporal graph definition.

A set of stations is defined as $\text{S}=\{{\text{S}}_{\text{i}}{\}}_{\text{i}=\text{1}}^{{\text{N}}_{\text{station}}}$ and a set of station groups is defined as $\text{G}=\{{\text{g}}_{\text{j}}{\}}_{\text{j}=\text{1}}^{{\text{N}}_{\text{group}}}$, where ${\text{N}}_{\text{station}}$ indicates the total number of stations and ${\text{N}}_{\text{group}}$ reflects the total number of station groups. A station graph is associated with a specific number of station groups. A station graph $\text{g}=\{{\text{V}}_{\text{s}},{\text{A}}_{\text{s}},\text{D},\text{E}\}$ is utilized to represent the relationships among stations, where ${\text{V}}_{\text{s}}$ represents the collection of station nodes, ${\text{A}}_{\text{s}}$ refers to the set of edges, D is the list of node attributes, and E is the edge adjacency matrix. Similarly, a station group graph $\text{P}=\{{\text{V}}_{\text{g}},{\text{A}}_{\text{g}},\text{H},\text{T}\}$ is employed to represent the relationships among station groups, where ${\text{V}}_{\text{g}}$ includes the group nodes, ${\text{A}}_{\text{g}}$ represents the list of edges among these groups, H stands for the matrix of group node attributes, and T depicts the matrix of edge attributes.

#### Multi-scale temporal modeling.

PM2.5 concentrations exhibit patterns across multiple temporal scales. Given the current time t₀ and prediction horizon Tₚ, we extract three complementary time segments: For recent observations, Xₕ∈ ${\text{R}}^{{\text{N}}_{\text{station}}\times \text{F}\times {\text{T}}_{\text{h}}}$ Captures short-term fluctuations from the past Tₕ hours. For Daily patterns, X_d_∈${\text{R}}^{{\text{N}}_{\text{station}}\times \text{F}\times \text{D}\times {\text{T}}_{\text{p}}}$ Extracts same-hour data from past D days to model diurnal cycles. For Weekly patterns, X_w_∈${\text{R}}^{{\text{N}}_{\text{station}}\times \text{F}\times \text{W}\times {\text{T}}_{\text{p}}}$ Samples same day-hour from past W weeks for weekly periodicity.

Here, N represents the number of monitoring stations and F denotes the feature dimension, comprising six pollutant measurements (PM2.5, PM10, NO₂, CO, O₃, SO₂) collected at each station. This multi-scale temporal decomposition allows the model to capture both immediate fluctuations and recurring patterns that characterize PM2.5 dynamics.

#### Prediction task.

Given historical observations at multiple scales {Xₕ, X_d_, X_w_} and station set S, we learn function f_θ_ to predict future PM2.5 concentrations:


$$\text{PM}{\hat{\text{2}}.\text{5}}^{\text{t}:\text{t}+\text{H}-\text{1}}={\text{f}}_{\theta }({\text{X}}_{\text{r}}{,{\text{X}}_{\text{d}},\text{X}}_{\text{w}},\text{S})$$ (1)

Here, θ represents the model parameters to be learned, and the predicted PM2.5 concentrations over the next Tₚ time steps are represented by $\text{PM}{\hat{\text{2}}.\text{5}}^{\text{t}:\text{t}+\text{H}-\text{1}}\in {\text{R}}^{{\text{N}}_{\text{station}}\times {\text{T}}_{\text{p}}}$.

## Research methodology

### Overview of method

The MSDGNN architecture addresses three key challenges through an integrated approach. Multi-scale temporal modeling (hourly, daily, weekly components) captures dependencies across different time horizons. The station grouping module discovers spatial correlations beyond geographic proximity, particularly valuable for sparse monitoring networks. Adaptive graph convolution dynamically modulates these spatial relationships based on learned attention weights.

**Fig 1:**
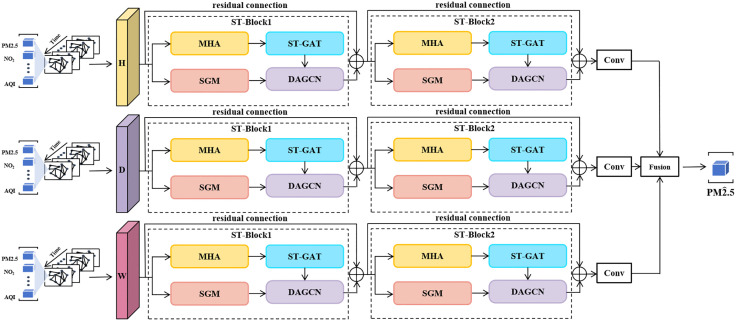
Schematic architecture of the proposed the MSDGNN (MSDGNN). The model processes input data at hourly **(H)**, daily **(D)**, and weekly (W) temporal scales through parallel components. Each component consists of stacked Spatiotemporal Blocks (ST-Blocks) that incorporate Multi-Head Attention (MHA), a Spatiotemporal Graph Attention Network (ST-GAT), a Station Grouping Module (SGM), and a Dynamic Adaptive Graph Convolution Network (DAGCN). Outputs from each scale are fused to generate the final prediction. Residual connections are employed to facilitate training.

As illustrated in Fig 1, MSDGNN consists of three parallel components processing historical data at different timescales. Each component contains multiple sequentially connected spatiotemporal blocks (ST-Blocks), with residual connections between blocks to optimize training efficiency. A fully connected layer with feature fusion combines outputs from the three components to generate final predictions. Each ST-Block integrates three modules: a station grouping module, an attention enhancement module, and a dynamic adaptive graph convolution network.

### Station grouping learning module

The model captures dependencies at multiple spatial scales by considering stations and station groups across different temporal scales. According to atmospheric dispersion theory, PM2.5 concentrations exhibit spatial heterogeneity driven by emission sources, meteorological transport, and chemical transformations. These factors naturally partition monitoring stations into functional groups.

#### Station group dependency modeling.

We employ a learnable mapping matrix $\text{S}\in {\text{R}}^{{\text{N}}_{\text{station}}\times {\text{N}}_{\text{group}}}$ to dynamically discover these latent patterns, where each element S_i,j_ represents the probability of station i belonging to group j. The matrix is initialized using Xavier uniform initialization, with row-wise softmax normalization maintaining probabilistic constraints during training:


$${\text{S}}_{\text{i},\text{j}}=\text{exp}\left({\tilde{\text{S}}}_{\text{i},\text{j}}\right)/\sum {\mathrm{\backslash nolimits}}_{\text{k}=\text{1}}^{\text{k}}\text{exp}\left({\tilde{\text{S}}}_{\text{i},\text{j}}\right)$$ (2)

**Fig 2:**
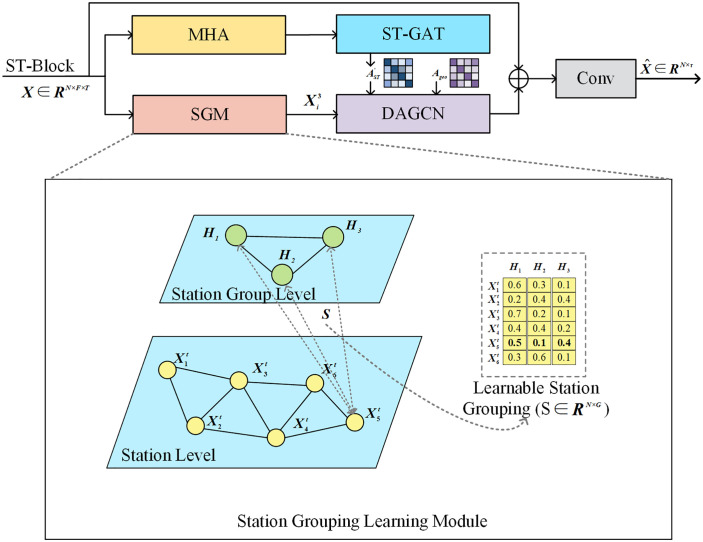
Station Grouping Learning Module (SGM) Architecture. The diagram depicts the hierarchical processing pipeline: input features X are processed by MHA and ST-GAT to compute dynamic attention, followed by the SGM which maps stations (yellow) to groups (green) via a learnable matrix S (see inset). Bidirectional information flow (dashed lines) and feature fusion via DAGCN enable the final prediction $\hat{𝐗}$.

The soft assignment is constrained so that the probabilities for each station sum to unity ($\sum_{\text{j}=\text{1}}^{\text{k}}{\text{S}}_{\text{i},\text{j}}$= 1). The weights in the matrix S thus reflect the relevance of each station to the different groups. A concrete example of this probability distribution for 6 stations across 3 groups is visualized in the inset table of Fig 2, which illustrates the hierarchical processing and bidirectional information flow of the Station Grouping Learning Module.

The station group representation ${\text{H}}_{\text{j}}$ for group ${\text{g}}_{\text{j}}$, is computed as:


$${\text{H}}_{\text{j}}=\sum {\mathrm{\backslash nolimits}}_{\text{i}=\text{1}}^{{\text{N}}_{\text{station}}}\;{\text{S}}_{\text{j},\text{i}}^{\text{T}}{\text{X}}_{\text{i}}^{\text{t}}$$ (3)

Here, ${\text{X}}_{\text{i}}^{\text{t}}$ represents the air quality data of station S_i._ This formulation enables stations with similar pollution patterns to be assigned to the same group, creating virtual monitoring regions that transcend geographical boundaries. After obtaining H_j_ for each station group, the dependencies among station groups are further computed, and temporal information is introduced to form more dynamic spatial dependencies. The edge attributes T_i,j_, between station group H_i_ and H_j_ are computed as:


$${\text{T}}_{\text{i},\text{j}}=\text{ReLU}\left(\text{MLP}\left({\text{H}}_{\text{i}},{\text{H}}_{\text{j}},\text{time}\right)\right)$$ (4)

The temporal encoding time ∈ R^6^ captures temporal features through sinusoidal functions that preserve natural periodicity, incorporating sine and cosine transformations of the hour of day (h ∈ [0,23], encoded as sin(2πh/24) and cos(2πh/24)), day of week (d ∈ [0,6], encoded as sin(2πd/7) and cos(2πd/7)), and month of year (m ∈ [1,12], encoded as sin(2πm/12) and cos(2πm/12)). The MLP leverages these encoded temporal patterns to dynamically modulate inter-group connections, strengthening connections from industrial zones during weekday working hours when PM2.5 transport is typically high, while weakening them during weekend nights when industrial emissions reduce. This temporal modulation effectively captures time-varying inter-group influences that reflect changing pollution transport patterns driven by diurnal boundary layer dynamics and seasonal variations.

Based on these dependencies, message aggregation and representation updates are performed. The message-passing mechanism comprises two main steps:


$$\begin{array}{c} {\text{T}}_{\text{i}}={\left\{\left({\text{H}}_{\text{i}},{\text{H}}_{\text{j}},{\text{T}}_{\text{j},\text{i}}\right)\right\}}_{\text{j}\neq \text{i}}\\ {\text{r}}_{\text{i}}\leftarrow \rho_{\text{g}}\left({\text{T}}_{\text{i}}\right)\\ {\text{H}}_{\text{i}}^{\prime }\leftarrow \varphi_{\text{g}}\left({\text{r}}_{\text{i}},{\text{H}}_{\text{i}}\right) \end{array}$$ (5)

Here, ${\text{H}}_{\text{i}}^{\prime }$ represents the revised station group structure, r_i_ denotes the aggregated from neighboring groups, and $\rho_{\text{g}}$ and ϕ_g_ represent the message aggregation and representation update functions, respectively, typically implemented by MLPs.

#### Station dependency modeling.

The updated station group representation ${\text{H}}_{\text{i}}^{\prime }$, which encodes regional pollution patterns, are then propagated back to individual stations to enrich local measurements with broader contextual information. Specifically, the representation of each station is refined by integrating information from all groups to which it belongs, weighted by the soft-assignment probabilities from matrix S:


$${\text{X}}_{\text{i}}^{\text{1}}=\sum {\mathrm{\backslash nolimits}}_{\text{j}=\text{1}}^{{\text{N}}_{\text{group}}}\;{\text{S}}_{\text{i},\text{j}}{\text{H}}_{\text{i}}^{\prime }$$ (6)

This step, ${\text{X}}_{\text{i}}^{\text{1}}$ represents an intermediate station representation fused with group-level insights. The final station representation is obtained through an additional message-passing step that incorporates information from neighboring stations, capturing both direct influences and complex correlations:


$$\begin{array}{c} {\text{X}}_{\text{i}}^{\text{2}}=\text{cat}\left({\text{X}}_{\text{i}},{\text{X}}_{\text{i}}^{\text{1}}\right)\\ {\text{T}}_{\text{i}}={\left\{\left({\text{X}}_{\text{i}}^{\text{2}},{\text{X}}_{\text{n}}^{\text{2}},{\text{E}}_{\text{n},\text{i}}\right)\right\}}_{\text{n}\in \text{n}\left(\text{i}\right)}\\ {\text{r}}_{\text{i}}\leftarrow \rho \left({\text{T}}_{\text{i}}\right)\\ {\text{X}}_{\text{i}}^{\text{3}}\leftarrow \varphi \left({\text{X}}_{\text{i}}^{\text{2}},{\text{r}}_{\text{i}}\right) \end{array}$$ (7)

Here, ${\text{E}}_{\text{n},\text{i}}$ represents the edge attributes between station group n and i, ${\text{T}}_{\text{i}}$ represents the collection of all messages sent to station ${\text{S}}_{\text{i}}$ from its neighbors, and ${\text{X}}_{\text{i}}^{\text{3}}$ is the final updated representation. This hierarchical, bidirectional information flow—upward aggregation from stations to groups and downward refinement from groups to stations—enables the model to effectively integrate multi-scale spatial dependencies for accurate prediction.

### Attention enhancement module

The attention enhancement module captures complex spatiotemporal dependencies through two complementary mechanisms: global spatial attention via Multi-Head Self-Attention (MSA) and dynamic spatiotemporal attention that adapts to temporal variations. These mechanisms work together to identify both long-range dependencies and time-varying patterns in PM2.5 propagation.

#### Spatial multi-head self-attention.

MSA captures global-scale spatial dependencies beyond local graph neighborhoods. Specifically, given the air quality data ${\text{X}}_{\text{i}}^{\text{t}}\in {\text{R}}^{\text{T}\times \text{N}\times \text{C}}$, MSA generates multiple subspaces via linear transformations, producing ${\text{Q}}_{\text{i}}^{\text{T}}\in {\text{R}}^{\text{N}\times {\text{d}}_{\text{K}}},{\text{K}}_{\text{i}}^{\text{T}}\in {\text{R}}^{\text{N}\times {\text{d}}_{\text{K}}},$ and ${\text{V}}_{\text{i}}^{\text{T}}\in {\text{R}}^{\text{N}\times {\text{d}}_{\text{M}}}$. They are computed as: ${\text{Q}}_{\text{i}}^{\text{S}}={\text{X}}_{\text{i}}^{\text{t}}{\text{W}}_{{\text{q}}_{\text{i}}}^{\text{S}},{\text{K}}_{\text{i}}^{\text{S}}={\text{X}}_{\text{i}}^{\text{t}}{\text{W}}_{{\text{k}}_{\text{i}}}^{\text{S}},{\text{V}}_{\text{i}}^{\text{S}}={\text{X}}_{\text{i}}^{\text{t}}{\text{W}}_{{\text{V}}_{\text{i}}}^{\text{S}}$. where ${\text{W}}_{{\text{q}}_{\text{i}}}^{\text{S}},{\text{W}}_{{\text{k}}_{\text{i}}}^{\text{S}}$, and ${\text{W}}_{{\text{V}}_{\text{i}}}^{\text{S}}$ are learnable parameters. Then, the spatial attention weights are calculated via a scaled dot-product operation:


$$\text{MSA}({\text{X}}_{\text{i}}^{\text{t}})=\text{Concat}\left({\text{head}}_{\text{1}}^{\text{S}},{\text{head}}_{\text{2}}^{\text{S}},\ldots ,{\text{head}}_{\text{n}}^{\text{S}}\right){\text{W}}^{\text{s}}\text{where}{\text{head}}_{\text{i}}^{\text{S}}=\text{Softmax}(\frac{{\text{Q}}_{\text{i}}^{\text{S}}({{\text{K}}_{\text{i}}^{\text{S}})}^{\text{T}}}{\sqrt{{\text{d}}_{\text{K}}}}){\text{V}}_{\text{i}}^{\text{S}}$$ (8)

Here, ${\text{W}}^{\text{s}}$ are learnable parameters. This mechanism identifies dependencies between distant monitoring stations that may share similar emission sources or meteorological patterns despite geographical separation.

#### Dynamic spatiotemporal graph attention mechanism.

After processing by MSA, the dynamic spatiotemporal attention mechanism can further refine the broad spatial relationships captured, particularly adjusting and optimizing time-sensitive changes. Specifically, spatiotemporal attention matrices are generated from dynamic temporal and spatial data and are employed to adjust dependencies among stations across various temporal scales and spatial ranges.

The temporal attention matrix A_T_ is calculated as:


$${\text{A}}_{\text{T}}={\text{W}}_{\text{a}}∙\sigma ((({\text{X}}_{\text{h}}^{\left(\text{r}-\text{1}\right)}{)}^{\text{T}}\Theta_{\text{1}})\Theta_{\text{2}}(\Theta_{\text{3}}{\text{X}}_{\text{h}}^{\left(\text{r}-\text{1}\right)})+\Upsilon_{\text{a}}){\text{A}}_{\text{T}}^{\prime }=\frac{\text{exp}({𝐀}_{\text{T}\text{i},\text{j}})}{\sum_{\text{j}=\text{1}}^{{\text{T}}_{\text{r}-\text{1}}}\text{exp}({𝐀}_{\text{T}\text{i},\text{j}})}$$ (9)

Here, $\Theta_{\text{1}},\Theta_{\text{2}},\Theta_{\text{3}}$ are learnable parameters, and ${\text{X}}_{\text{h}}^{\left(\text{r}-\text{1}\right)}\in {\text{R}}^{\text{N}\times \text{T}\times \text{C}}$ is the MSA output, representing monitoring station features at iteration r-1. Similarly, the spatial attention matrix Satt is computed as:


$${\text{A}}_{\text{S}}={\text{W}}_{\text{b}}∙\sigma \left({\text{X}}_{\text{h}}^{\left(\text{r}-\text{1}\right)}\phi_{\text{1}}\right)\phi_{\text{2}}\left((\phi_{\text{3}}{\text{X}}_{\text{h}}^{\left(\text{r}-\text{1}\right)}{)}^{\text{T}}+{\text{b}}_{\text{s}}\right){\text{A}}_{\text{S}\text{i},\text{j}}^{\prime }=\frac{\text{exp}({\text{A}}_{\text{S}\text{i},\text{j}})}{\sum_{\text{j}=\text{1}}^{\text{N}}\text{exp}({\text{A}}_{\text{S}\text{i},\text{j}})}$$ (10)

Here, $\phi_{\text{1}},\phi_{\text{2}},\phi_{\text{3}}$ are learnable parameters, ${\text{W}}_{\text{b}}$ denotes the weight matrix, ${\text{b}}_{\text{s}}$ represents the bias term, and ${\text{X}}_{\text{h}}^{\left(\text{r}-\text{1}\right)}$ represents the information integrated with temporal attention. By integrating A_T_ and A_S_, the model can comprehensively consider dynamic changes in both time and space.

### Dynamic adaptive graph convolution network

The DAGCN propagates features across the station network. DAGCN combines an adaptive adjacency matrix--capturing persistent spatial relationships between stations--with dynamic attention weighting that adjusts information flow based on current conditions. The following section first describes the graph construction method, followed by an explanation of the attention-modulated spectral graph convolution operation.

#### Adaptive adjacency matrix.

We construct two complementary adjacency matrices. The geographical adjacency matrix A_geo_ derives from monitoring station distances, where A_geo_[i,j] = 1 if the Euclidean distance between stations i and j is within 18 km, and 0 otherwise, reflecting direct spatial proximity and local pollutant transport between neighboring stations. To capture latent dependencies beyond physical proximity, we learn an adaptive adjacency matrix Ã_adp_ through node embeddings E_1_, E_2_ ∈ R^N×d^:


$${\tilde{\text{A}}}_{\text{adp}}=\text{SoftMax}(\text{ReLU}({\text{E}}_{\text{1}}{\text{E}}_{\text{2}}^{\text{T}}))$$ (11)

This learned matrix identifies hidden correlations such as stations sharing similar emission sources or meteorological patterns, optimized through end-to-end training. The node embeddings E_1_ and E_2_ are learned during training and remain fixed during inference, capturing persistent spatial correlations between stations. The hybrid graph combines both matrices:


$${\text{A}}_{\text{hybrid}}=\text{a}\times {\text{A}}_{\text{geo}}+\left(\text{1}-\alpha \right)\times {\tilde{\text{A}}}_{\text{adp}}$$ (12)

Here, α∈ [0,1] is a learnable parameter balancing geographical priors with data-driven patterns. This approach proves particularly valuable for sparse monitoring networks.

#### Chebyshev polynomial graph convolution.

To avoid direct eigenvector decomposition of the Laplacian matrix and reduce computational complexity, we use Chebyshev polynomials to approximate the convolution kernel:


$${\text{g}}_{\theta }*\text{Gx}=\theta_{\text{k}}{\text{P}}_{\text{k}}^{\prime }\text{x}$$ (13)

In this context, $\theta_{\text{k}}\in {\text{R}}^{\text{K}}$ are learnable parameters, and ${\text{P}}_{\text{k}}^{\prime }$ represents the k-th order Chebyshev polynomial matrix. The Chebyshev polynomials matrices are computed through an efficient recursive formulation:


$${\text{P}}_{\text{k}}^{\prime }=\text{2}{\text{A}}_{\text{hybrid}}∙{\text{P}}_{\text{k}-\text{1}}^{\prime }-{\text{P}}_{\text{k}-\text{2}}^{\prime }(\text{K}\geq \text{2})$$ (14)

Here, ${\text{P}}_{\text{0}}^{\prime }={\text{I}}_{\text{N}}{\text{and}\text{P}}_{\text{1}}^{\prime }={\text{A}}_{\text{bybrid}}$. I_N_ is the identity matrix and ${\text{A}}_{\text{hybrid}}$ is the hybrid adjacency matrix.

PM2.5 propagation patterns vary with meteorological conditions and temporal dynamics. We modulate the Chebyshev polynomial matrices with spatial attention computed from current input features:


$${\text{P}}_{\text{k}}^{\text{att}}={\text{P}}_{\text{k}}^{\prime }⨀{\text{A}}_{\text{S}}^{\prime }$$ (15)

Here, ⨀ denotes element-wise (Hadamard) product. This modulation allows the model to dynamically reweight the information flow between stations at each time step, enhancing the importance of connections critical under current conditions (e.g., wind-driven transport).

Finally, convolution is employed to capture the evolving trends of PM2.5 concentrations over varying time periods. The complete formula is formulated as:


$${\text{X}}_{\text{h}}^{\left(\text{r}\right)}=\text{ReLU}\left({\text{W}}_{\text{learn}}\left(\text{ReLU}\left(\theta_{\text{k}}{\text{P}}_{\text{k}}^{\text{att}}{\hat{\text{X}}}_{\text{h}}^{\left(\text{r}-\text{1}\right)}\right)\right)\right)$$ (16)

Here, ${\text{W}}_{\text{learn}}$ represents learnable weight parameters, ${\hat{\text{X}}}_{\text{h}}^{\left(\text{r}-\text{1}\right)}$denotes the cross-city structure-enhanced features obtained from the station grouping module at iteration r-1, and ${\text{P}}_{\text{k}}^{\text{att}}$ aggregates information from k-hop neighborhoods with attention-modulated weights.

### Multi-Component fusion

To comprehensively process features at various spatiotemporal scales, a multi-component fusion strategy is employed in this study. By weighting and combining near-hour, daily, and weekly features, multi-scale information integration is achieved. The fusion formula is given by:


$$\text{PM}\hat{\text{2}}.\text{5}={\text{Q}}_{\text{h}}⨀{\text{PM}\hat{\text{2}}.\text{5}}_{\text{h}}+{\text{Q}}_{\text{d}}⨀{\text{PM}\hat{\text{2}}.\text{5}}_{\text{d}}+{\text{Q}}_{\text{w}}⨀{\text{PM}\hat{\text{2}}.\text{5}}_{\text{w}}$$ (17)

Here, ${\text{Q}}_{\text{h}}$, ${\text{Q}}_{\text{d}}$, ${\text{Q}}_{\text{w}}$ are the weights of each component, and ${\text{PM}\hat{\text{2}}.\text{5}}_{\text{h}}$, ${\text{PM}\hat{\text{2}}.\text{5}}_{\text{d}}$, ${\text{PM}\hat{\text{2}}.\text{5}}_{\text{w}}$ are the predicted values for the short-term, daily cycle, and weekly cycle components, respectively.

### Model training and evaluation metrics

The model was trained with a fixed Chebyshev polynomial order and temporal convolution kernel size of 3. Each graph convolutional layer and temporal convolutional layer employed 64 filters. The GNN architecture consisted of two layers with a hidden dimension of 32. The Mean Squared Error (MSE) between predictions and ground truth was used as the loss function, optimized via backpropagation with a batch size of 32 and a learning rate of 0.001.

Hyperparameters were determined through grid search on the validation set, with search ranges and selected values presented in Table 1.

**Table 1:** Hyperparameter Search Ranges and Selected Values.

Hyperparameter	Search Range	Selected Value
Learning rate	[0.0001,0.005]	0.001
Batch size	{16,32,64}	32
Hidden dimension	{32,64,128}	64
GNN layers	{1,2,3}	2
Station groups(K)	{2,3,4,5,6,7,8,9}	3
Chebyshev polynomial order	{2,3,4}	3
Dropout rate	{0.1,0.2,0.3}	0.2

The selected configuration balances model capacity and computational efficiency, achieving optimal validation performance while mitigating underfitting and overfitting risks.

The dataset was split chronologically into training, validation, and testing sets with a ratio of 60:20:20. This partitioning strategy preserves temporal dependencies, prevents data leakage, and simulates real-world forecasting scenarios. The 60% training set facilitates effective learning of multi-scale temporal patterns, while the 20% validation and test sets enable robust evaluation across seasonal variations. This approach aligns with established practices in spatiotemporal forecasting literature [26] and ensures comparability with state-of-the-art methods.

All implementations were based on PyTorch [32], with experiments conducted on a server equipped with an NVIDIA RTX 3090 GPU.

To evaluate the performance of various models from multiple angles, four metrics are selected: Mean Absolute Error (MAE), Root Mean Squared Error (RMSE), Weighted Absolute Percentage Error (WAPE), and Correlation Coefficient (CORR). These measures provide comprehensive insights into the predictive accuracy and effectiveness of the models. The corresponding formulas are as follows:


$$\text{MAE}=\frac{\text{1}}{{\text{N}}_{\text{city}}}\sum {\mathrm{\backslash nolimits}}_{\text{i}=\text{1}}^{{\text{N}}_{\text{city}}}|\text{PM2}.{\text{5}}_{\text{i}}^{\text{t}+\text{k}}-\text{PM}\hat{\text{2}}.{\text{5}}_{\text{i}}^{\text{t}+\text{k}}|$$ (18)

$$\text{WAPE}=\frac{\text{100}\%}{{\text{N}}_{\text{city}}}\frac{\sum_{\text{i}=\text{1}}^{{\text{N}}_{\text{city}}}|\text{PM2}.{\text{5}}_{\text{i}}^{\text{t}+\text{k}}-\text{PM}\hat{\text{2}}.{\text{5}}_{\text{i}}^{\text{t}+\text{k}}|}{\sum_{\text{i}=\text{1}}^{{\text{N}}_{\text{city}}}\text{PM2}.{\text{5}}_{\text{i}}^{\text{t}+\text{k}}}$$ (19)

$$\text{RMSE}=\sqrt{\frac{\text{1}}{{\text{N}}_{\text{city}}}\sum {\mathrm{\backslash nolimits}}_{\text{i}=\text{1}}^{{\text{N}}_{\text{city}}}|\text{PM2}.{\text{5}}_{\text{i}}^{\text{t}+\text{k}}-\text{PM}\hat{\text{2}}.{\text{5}}_{\text{i}}^{\text{t}+\text{k}}{|}^{\text{2}}}$$ (20)

$$\text{CORR}=\text{1}-(\sum {\mathrm{\backslash nolimits}}_{\text{i}=\text{1}}^{{\text{N}}_{\text{city}}}|{\text{PM2}.{\text{5}}_{\text{i}}^{\text{t}+\text{k}}-\text{PM}\hat{\text{2}}.{\text{5}}_{\text{i}}^{\text{t}+\text{k}}|}^{\text{2}})/(\sum {\mathrm{\backslash nolimits}}_{\text{i}=\text{1}}^{{\text{N}}_{\text{city}}}|{\text{PM2}.{\text{5}}_{\text{i}}^{\text{t}+\text{k}}-\text{M}\hat{\text{2}}.{\text{5}}_{\text{i}}^{\text{t}+\text{k}}|}^{\text{2}})$$ (21)

### Statistical significance testing

All experimental results are reported as the average over five independent runs with different random seeds (42, 123, 256, 512, 1024) to ensure reliability. The statistical significance of performance differences—both for MSDGNN versus baseline models and for the complete model versus its ablation variants—was evaluated with paired t-tests (p < 0.001, *p < 0.01, *p < 0.05).

## Results and analysis

### Performance comparison with baselines

The proposed MSDGNN model was compared with seven established models—DGCRN [27], Crossformer [30], Autoformer [33], AGCRN [26], Informer [34], LSTM [35], and Historical Average(HA)— to evaluate their multi-step prediction capabilities on the PM2.5 test dataset. Predictive analyses were conducted for 16-hour, 24-hour, and 32-hour horizons. For the 32-hour prediction, models were required to forecast concentrations for the subsequent 32 time steps, with evaluation metrics averaged across all points from hour 1 to hour 32. Table 2 presents the comparative performance, where the best result for each metric is highlighted in bold.

**Table 2:** Performance Comparison of Different Models.

Method		16h				24h				32h		
	**MAE**	**RMSE**	**WAPE**	**CORR**	**MAE**	**RMSE**	**WAPE**	**CORR**	**MAE**	**RMSE**	**WAPE**	**CORR**
**HA**	11.8202^***^	17.0311^***^	0.4206^***^	0.5674^***^	11.8563^***^	16.9014^***^	0.4221^***^	0.5702^***^	13.6808^***^	19.0925^***^	0.4868^***^	0.4577^***^
**LSTM**	9.4297^***^	12.7409^*^	0.2999	0.7050	9.7007^***^	14.4443^**^	0.3468^**^	0.6606^*^	10.6424^***^	15.7375^***^	0.3781^***^	0.6097^***^
**AGCRN**	10.7036^***^	15.2739^***^	0.3809^***^	0.5925^***^	11.2018^***^	15.7350^***^	0.3987^***^	0.5528^***^	11.5361^***^	16.0474^***^	0.4105^***^	0.4812^***^
**DGCRN**	9.0203^***^	13.5575^***^	0.3212^***^	0.7123	12.5436^***^	17.2019^***^	0.4464^***^	0.6727^**^	14.2326^***^	18.6801^***^	0.5066^***^	0.6397
**Crossformer**	9.7674^***^	14.4277^***^	0.3298^***^	0.6613^***^	9.2675^*^	13.9764	0.3476	0.6304^***^	10.1619^***^	14.8287^***^	0.3616^*^	0.5025^***^
**Informer**	10.1798^***^	14.7321^***^	0.3622^***^	0.6139^***^	10.7248^***^	15.3133^***^	0.3817^***^	0.5772^***^	11.2707^***^	15.9321^***^	0.4010^***^	0.5391
**Autoformer**	14.7906^***^	19.7434^***^	0.4657^***^	0.6071^***^	12.1634^***^	16.6586^***^	0.4329^***^	0.5877^***^	12.9966^***^	17.6139^***^	0.4625^***^	0.5406^***^
**MSDGNN**	**8.3658**	**12.2506**	**0.2999**	**0.7351**	**9.1223**	**13.2432**	**0.3274**	**0.6978**	**9.4742**	**13.5433**	**0.3397**	**0.6612**

As shown in Table 2, MSDGNN achieved superior performance across all evaluation metrics and prediction horizons. Specifically, for the 32-hour prediction, MSDGNN reduced MAE and RMSE by 6.77% and 8.67%, respectively, compared to the second-best model, Crossformer. Corresponding improvements of 10.17% in WAPE and 8.45% in CORR further underscore MSDGNN’s strong adaptability to sparse-site data. This consistent advantage stems from the model’s enhanced capability to efficiently capture multi-scale spatiotemporal features, thereby improving the representation of intricate dependencies.

**Fig 3:**
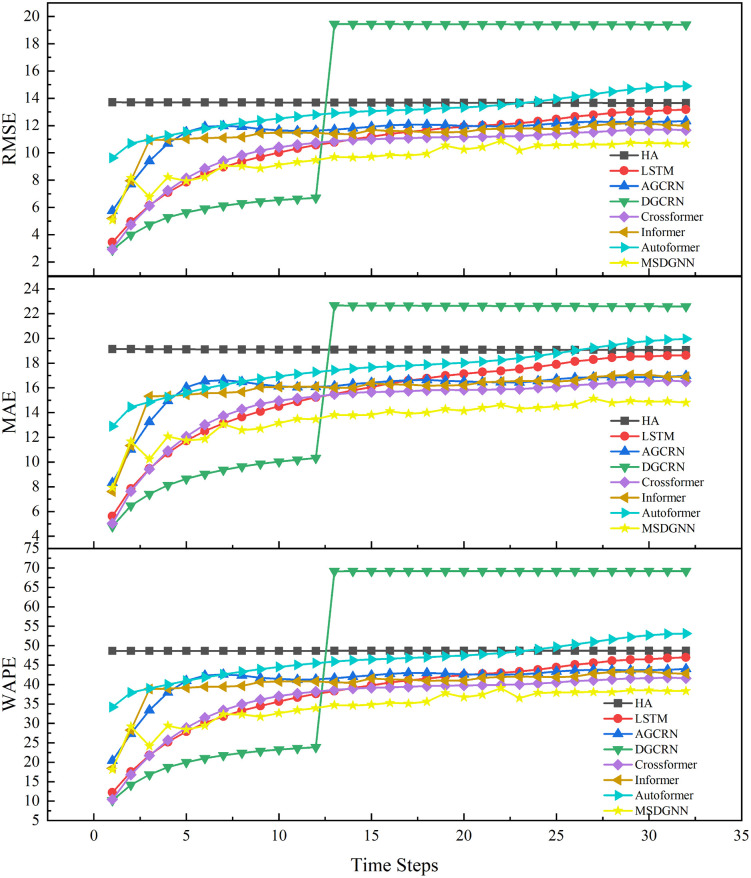
Trends of Evaluation Metrics Over Multiple Time Steps. Fig 3 illustrates the performance of MSDGNN compared to other baseline models across different prediction horizons using RMSE, MAE, and WAPE metrics. The three vertically arranged subplots depict the error rate trends over 30 time steps for each model. The results demonstrate that MSDGNN (green line) maintains consistently lower and more stable error rates throughout the entire prediction period.

To evaluate performance stability across forecasting periods, Fig 3 illustrates the RMSE, MAE, and WAPE metrics for MSDGNN and baseline models. Throughout the prediction horizon, MSDGNN exhibited more stable and accurate prediction capabilities. In contrast, HA maintained consistently high errors, as expected from a simple baseline that ignores temporal and spatial dependencies. DGCRN showed relatively strong performance for horizons shorter than 12 hours, but its error increased sharply beyond this point, revealing limitations in modeling long-term dependencies. Similarly, LSTM and Autoformer exhibited marked performance degradation at longer horizons, indicating a failure to fully capture long-range spatiotemporal correlations. MSDGNN, by comparison, maintained low error rates across both short and long horizons, reflecting its robust ability to model complex relationships. Its superior error control, particularly when handling multi-scale features and sparse-site data, demonstrates greater robustness and adaptability, making it a more reliable solution for complex PM2.5 concentration forecasting.

**Fig 4:**
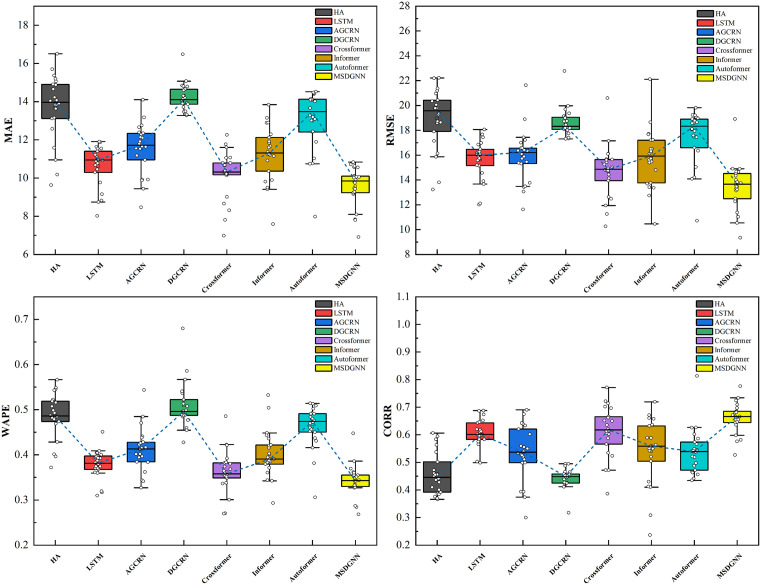
Comparative Prediction Performance Across Multiple Stations. Fig 4 presents the error distributions of different models at the site level through boxplots for four evaluation metrics (MAE, RMSE, WAPE, and CORR). The results demonstrate that MSDGNN (yellow) exhibits lower median values and interquartile ranges with fewer outliers for MAE, RMSE, and WAPE metrics, while achieving higher values for the CORR metric.

We further evaluated model performance at the site level using boxplots of the error distributions for each station, as shown in Fig 4. For MAE and RMSE, MSDGNN exhibited lower medians, first and third quartiles, and fewer outliers compared to other models, indicating smaller overall prediction errors and higher stability across the monitoring network. The narrower interquartile range further confirms the consistency of its predictions. For scale-invariant metrics, MSDGNN achieved a higher median CORR and a lower median WAPE, reflecting a stronger correlation between predictions and observations, as well as higher overall accuracy. In contrast, models like Crossformer and Informer showed higher error medians and greater dispersion, suggesting limitations in handling the spatiotemporal complexities of PM2.5 data. While AGCRN and DCRNN exhibited more concentrated error distributions, their accuracy remained lower than that of MSDGNN. LSTM and HA produced the widest boxplots and most dispersed error distributions, resulting in larger, more unstable prediction errors. These results can be attributed to MSDGNN’s effective capture of multi-scale spatiotemporal characteristics and its use of an adaptive graph convolution module, which enhances the model’s adaptability to data from sparsely distributed stations.

**Fig 5:**
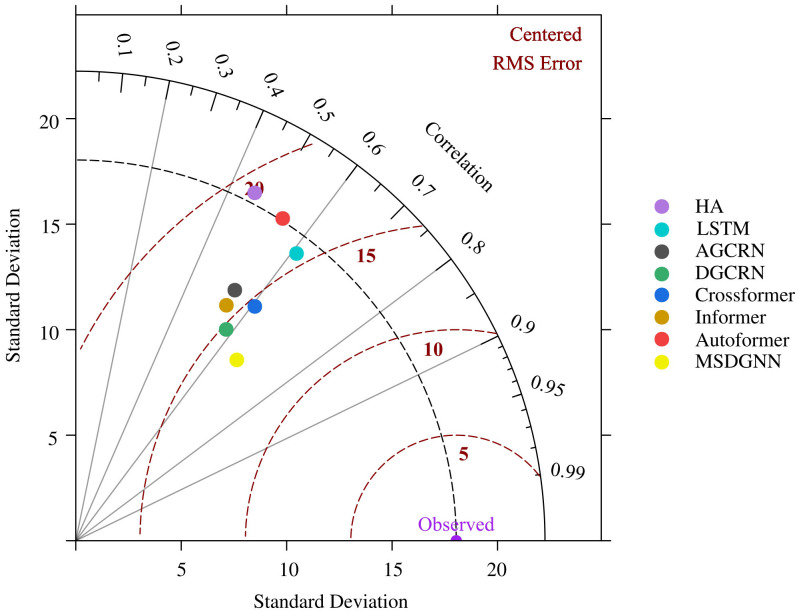
Model Performance Evaluation Using Taylor Diagram. Fig 5 compares the standard deviation, correlation coefficient, and centered root-mean-square error of predictions from different models against observations. The proximity of MSDGNN point (yellow) to the observed reference point (purple) indicates its superior performance.

To visually summarize the comprehensive performance, a Taylor diagram was employed in Fig 5, incorporating standard deviation, correlation coefficient, and centered root-mean-square error (RMSE). MSDGNN showed the closest alignment with observed values, with a correlation coefficient near 0.7, a standard deviation most consistent with observations, and the smallest centered RMSE. These findings highlight MSDGNN’s superior ability to capture both the trends and variability of PM2.5 concentrations with minimal error.

### Ablation studies

Ablation studies were conducted to assess the contribution of each key module within the MSDGNN framework. The results are summarized in Table 3, where the best performance for each evaluation metric is highlighted in bold.

**Table 3:** Impact of Key Modules on Overall Model Performance.

Model Variant	16h				24h				32h			
	MAE	RMSE	WAPE	CORR	MAE	RMSE	WAPE	CORR	MAE	RMSE	WAPE	CORR
**w/o MHA**	8.4246^*^	12.2812	0.3021^**^	0.7328	9.1811	13.0726^***^	0.3291^**^	0.6971	9.7090^***^	13.7264^**^	0.3527^***^	0.6388^***^
**w/o A**_**adp**_	8.6761^***^	12.6568^***^	0.3111^***^	0.7306	9.4658^***^	13.7283^***^	0.3393^***^	0.6906^*^	9.8418^***^	14.0058^***^	0.3476^***^	0.6578^*^
**w/o SGM**	8.5715^***^	12.5623^***^	0.3074^***^	0.7238^***^	9.3368^***^	13.4945^***^	0.3347^***^	0.6821^***^	9.6494^***^	13.8236^***^	0.3458^**^	0.6514^*^
**w/o Time**	8.3736	12.2864	0.2989	0.7316^*^	9.1841	13.1814^**^	**0.3256**	0.6885^**^	9.7426^***^	13.7137^*^	0.3475^***^	0.6486^***^
**MSDGNN**	**8.3658**	**12.2506**	**0.2999**	**0.7351**	**9.1223**	**13.2432**	0.3270	**0.6978**	**9.4742**	**13.5433**	**0.3397**	**0.6612**

**Fig 6:**
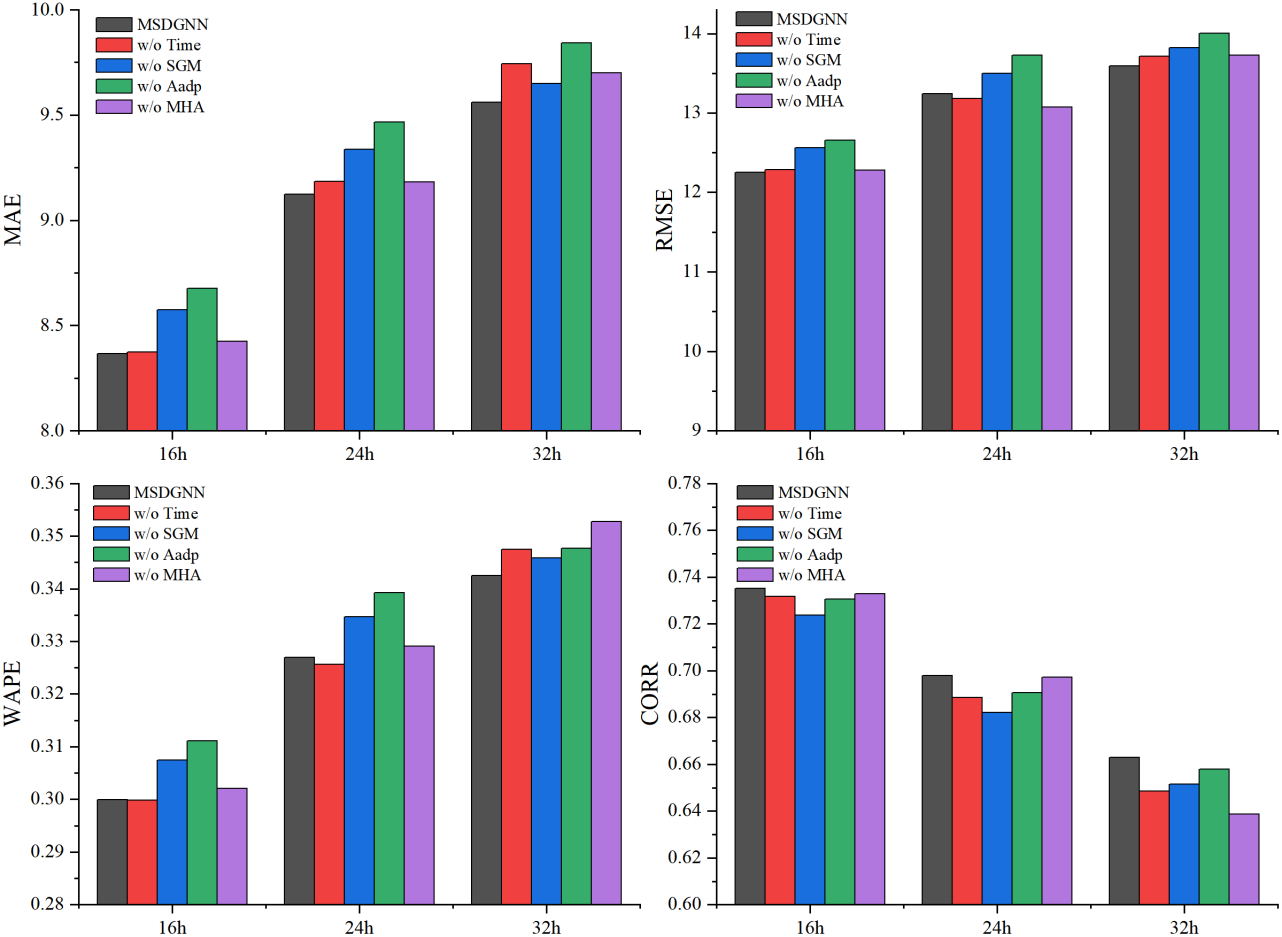
Comparison of Ablation Experiment Results. Fig 6 illustrates the performance differences between the complete MSDGNN model and its ablation variants (without multi-head attention: w/o MHA; without adaptive module: w/o A_adp_; without station grouping module: w/o SGM; without time component: w/o Time) across 16-hour, 24-hour, and 32-hour prediction horizons.

For comparison, the performance of each ablation model is also plotted in Fig 6. The results demonstrate that the complete MSDGNN model outperforms all ablation variants across all evaluation metrics and prediction horizons.

Performance declined markedly upon the removal of any major component. Eliminating the Multi-Head Attention (MHA) module led to an increase in MAE by approximately 15.94%, 18.19%, and 18.11% for the 16-hour, 24-hour, and 32-hour predictions, respectively, confirming its critical role in modeling long-range spatial relationships, which is crucial for long-term prediction accuracy under sparse-site data conditions. The most significant degradation occurred when the adaptive module (A_adp_) was removed, which resulted in an approximately 23.82% increase in RMSE for the 24-hour forecast, demonstrating its importance in capturing dynamic spatiotemporal variations. Without the Station Grouping Module (SGM), CORR values decreased by about 1.62%, 2.00%, and 2.16% for the respective horizons, indicating its essential role in capturing spatial dependencies and enabling effective interactions among sites. While the removal of the time component only slightly affected short-term WAPE (a 0.29% reduction at 16 hours), it caused substantial performance drops in long-term predictions, with MAE increasing by 19.19% and CORR decreasing by 2.00% at the 32-hour horizon, underscoring its necessity for modeling long-term temporal dependencies. In conclusion, the complete model provides more accurate predictions across all time horizons, validating the synergistic contribution of each integrated module.

### Analysis of the station grouping mechanism

The station grouping learning module significantly enhanced MSDGNN’s prediction performance. This module operates by learning soft assignments derived from station feature representations, rather than relying solely on geographic coordinates. This enables the identification of latent spatial structures that conventional distance-based approaches tend to overlook. Although the optimal number of groups may vary across datasets, the data-driven strategy established here offers a principled and geography-agnostic framework for modeling spatial dependencies in sparse monitoring networks.

**Fig 7:**
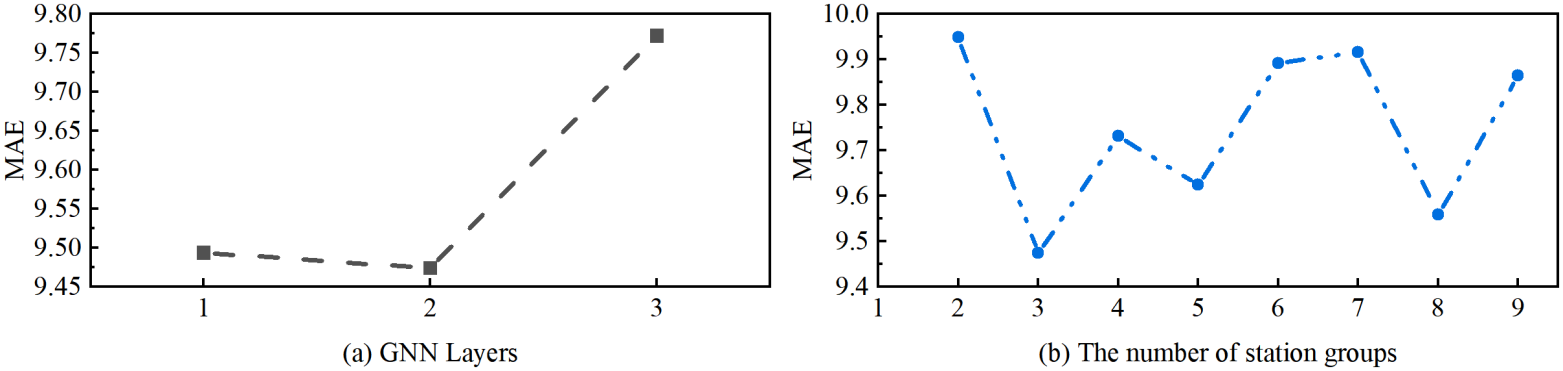
Evaluation Results for Different GNN Layers and Station Group Numbers. Subplot (a) demonstrates the impact of GNN layer count on model performance, indicating that a two-layer structure (MAE ≈ 9.48) outperforms both single-layer and three-layer configurations. Subplot (b) depicts the influence of station group quantity, revealing that the model achieves the lowest MAE (approximately 9.5) when the number of groups is set to three.

We analyzed two critical hyperparameters: the number of GNN layers and the number of station groups. As illustrated in Fig 7, performance was evaluated under different hyperparameter settings. Fig 7(a) indicates that a two-layer GNN structure achieved the best performance (MAE ≈ 9.48), balancing spatial feature extraction and model complexity. A single-layer model lacked sufficient expressive power, while a three-layer network introduced over-smoothing. Fig 7(b) shows that the model performed best when the number of station groups was set to three (MAE ≈ 9.5). This optimal grouping reflects the underlying functional regions within the monitoring network. Using fewer than three groups failed to distinguish key spatial patterns, whereas more groups led to diminishing returns—consistent with spatial complexity theory, where granularity must balance heterogeneity capture and overfitting risk.

**Fig 8:**
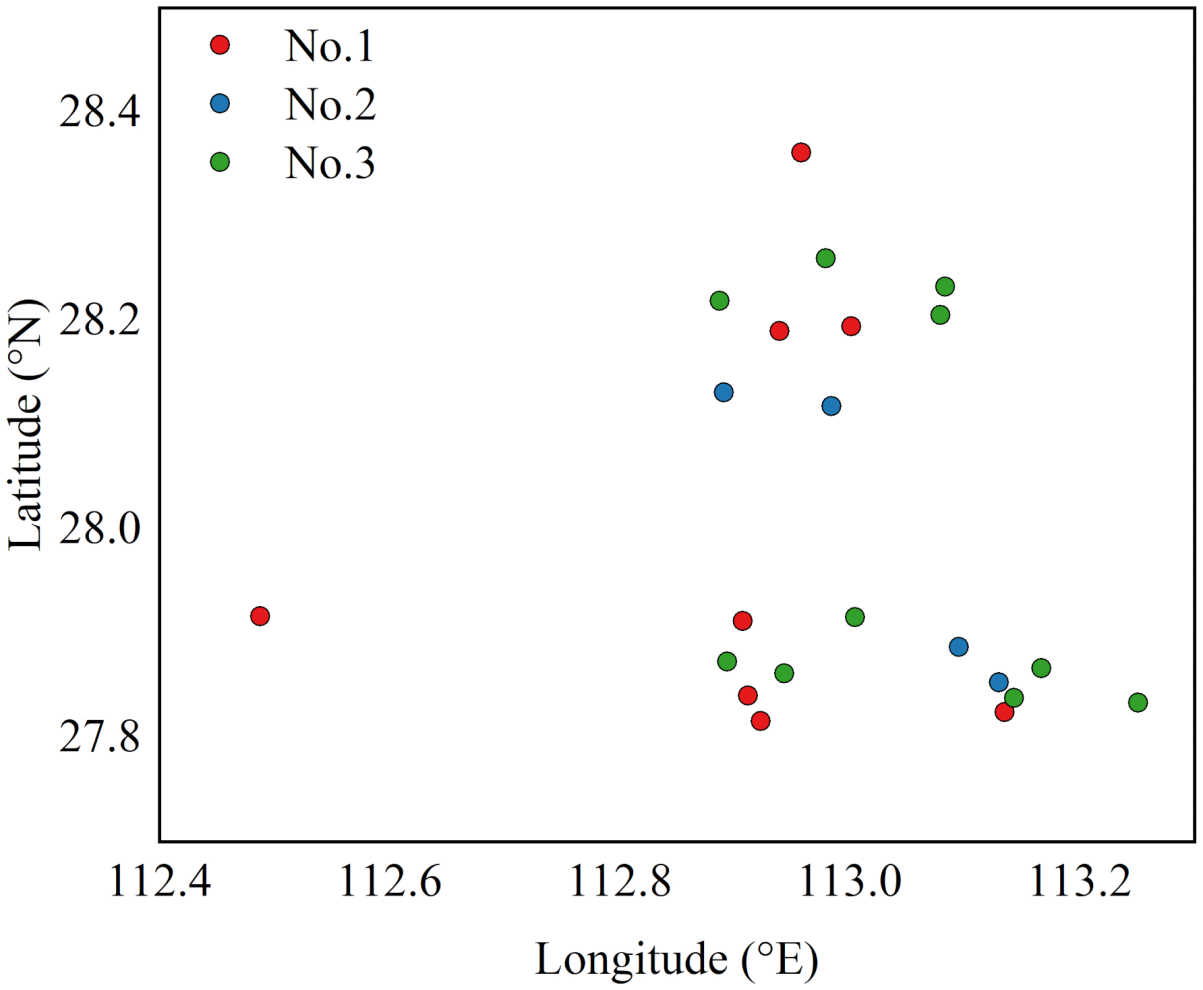
Results of Station Grouping. Fig 8 presents the visualization of station groupings in the Chang-Zhu-Tan region when the number of groups is set to three. The stations are plotted using their geographical coordinates (longitude and latitude), with each station differentiated by color: (Group No.1), blue (Group No.2), and green (Group No.3) colors, demonstrating a distribution based on functional similarity rather than geographical proximity. The spatial distribution of approximately 22 stations is displayed in coordinate space. For visualization clarity, each station is assigned to the group with the highest probability from the learned matrix **S.** However, during model training and inference, the full soft assignment probabilities are used in computations (as shown in Equations 3 and 6), allowing stations to partially belong to multiple groups with different membership weights.

Fig 8 visualizes the grouping result for the Chang-Zhu-Tan region, where each station is assigned to its most probable group based on the learned matrix S, distinguished by red (Group No.1), blue (Group No.2), and green (Group No.3). Stations are distributed across the three cities, with some clustered near the central river and others in peripheral areas.

As summarized in Table 4, the three groups exhibit distinct statistical characteristics. Group No.2 shows the highest variance (136.2) and prediction errors, yet maintains the strongest correlation (0.67), reflecting high variability but well-captured dynamics. Group No.3 is the most stable (Var = 119.1) and has the lowest errors, indicating higher prediction accuracy. Group No.1 falls between the two. These differences confirm that MSDGNN adaptively learns inherent data characteristics, enabling performance-optimized spatial grouping without predefined partitions.

**Table 4:** Performance metrics of station groups.

Group	Variance (Var)	MAE	RMSE	WAPE	CORR
**No.1**	125.0095	9.7044	13.815	34.0081	0.6511
**No.2**	136.2266	9.9904	14.0477	36.0751	0.6735
**No.3**	119.0526	9.0836	13.1242	33.1076	0.6643

### Case study: analysis of prediction results at individual stations

**Fig 9:**
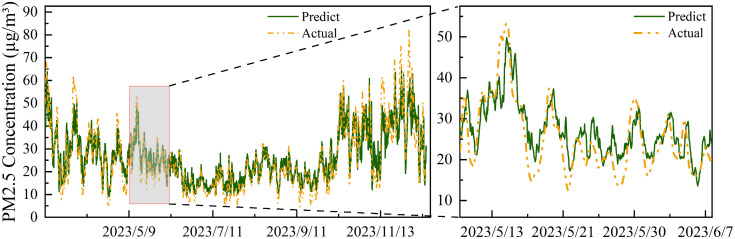
Prediction Results for Station 1339A (Best Performance). The left panel depicts long-term trends from May to November 2023, and the right panel shows a magnified view of short-term details from May 13 to June 7, 2023. The high degree of overlap between predictions (green solid lines) and actual observations (yellow dashed lines) demonstrates the model’s success in capturing periodic fluctuations and trend variations at both scales.

**Fig 10:**
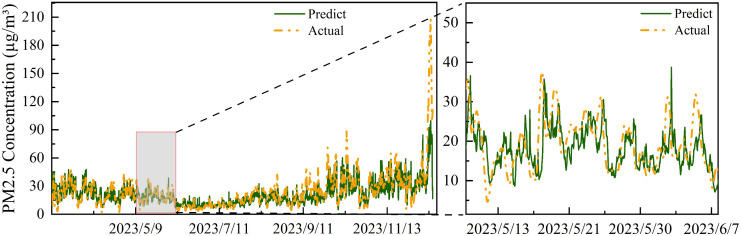
Prediction Results for Station 1559A (Worst Performance). Despite relatively lower overall prediction performance at this station, the model’s predictions (green solid lines) still effectively track the trend variations of actual observations (yellow dashed lines), particularly during periods of sharp concentration increases in November 2023. This demonstrates the model’s robust capability in modeling multi-scale spatiotemporal features at more challenging sites.

To gain deeper insights into the model’s performance, we selected Station 1339A, which exhibited the best MAE performance, and Station 1559A, which exhibited the worst. As shown in Figs 9 and 10, the model effectively captures periodicity and trends in the test set for both stations, demonstrating robust spatiotemporal modeling capabilities. This ability stems from the effective extraction of both local fluctuations and global trends in multi-scale spatiotemporal features.

## Conclusion

In this study, we proposed the Multi-Scale Dynamic Graph Neural Network (MSDGNN), a novel framework for predicting PM2.5 concentrations. By leveraging a multi-scale spatiotemporal modeling architecture, MSDGNN effectively captures complex dependencies at both global and local scales. Comprehensive evaluation on a real-world air quality pollutant dataset from the Chang-Zhu-Tan region demonstrates that our model maintains high predictive accuracy even with a sparse network of monitoring stations. This highlights MSDGNN’s significant advantages in handling the challenges posed by sparse station distribution and complex spatiotemporal correlations.

The MSDGNN framework serves as a robust and generalizable tool for spatiotemporal pattern extraction and forecasting. Its modular design ensures that it is not solely limited to PM2.5 prediction but also provides a foundational architecture for modeling a wide range of other spatiotemporal phenomena.

Future research will focus on extending the application of MSDGNN to other urban areas and further optimizing its architecture to enhance computational efficiency. We plan to employ adaptive methods, such as Fast Fourier Transform (FFT) analysis, for the automatic identification of dominant periods within time series data. This will allow for dynamic temporal scale selection, moving beyond the fixed hourly, daily, and weekly scales used in the current study. Such advancements are expected to improve the model’s adaptability and performance across diverse geographical and temporal contexts.
